# Highlights of Tuft Cells in Mouse and Human Salivary Glands

**DOI:** 10.3390/cells15070583

**Published:** 2026-03-25

**Authors:** Michael E. Rusiniak, Lara Shimagami, Victor Zanetti Drumond, Mariana Silveira Souza, Fernanda Luiza Araujo Lima de Castro, Chao Xue, Ming Zhang, Jun Qu, George Edward Chlipala, Mark Maienschein-Cline, Tarcilia Aparecida da Silva, Silvia Ferreira de Sousa, Harim Tavares dos Santos

**Affiliations:** 1 Department of Oral Biology, The State University of New York at Buffalo, Buffalo, NY 14214, USA; merusini@buffalo.edu (M.E.R.); larashim@buffalo.edu (L.S.); 2 Department of Oral Surgery, Oral Pathology and Clinical Dentistry, School of Dentistry, Federal University of Minas Gerais, Belo Horizonte 31270, Brazil; victorzanettiemc@gmail.com (V.Z.D.); marianasilveirasouza18@gmail.com (M.S.S.); fernandaluiza.alc@gmail.com (F.L.A.L.d.C.); tarcilia@ufmg.br (T.A.d.S.); silviafsousa@ufmg.br (S.F.d.S.); 3 New York State Center of Excellence in Bioinformatics & Life Sciences, Buffalo, NY 14203, USA; chaoxue@buffalo.edu (C.X.); mzhang25@buffalo.edu (M.Z.); junqu@buffalo.edu (J.Q.); 4 Research Informatics Core, Research Resources Center, University of Illinois Chicago, Chicago, IL 60607, USA; gchlip2@uic.edu (G.E.C.); mmaiensc@uic.edu (M.M.-C.)

**Keywords:** tuft cell, salivary gland, saliva, Sjögren’s, transcriptome and histology

## Abstract

**Highlights:**

**What are the main findings?**

- Loss of tuft cells is associated with sex-biased salivary gland remodeling in mice (transcriptome, histology, saliva flow/proteome).
- In human minor salivary glands, tuft cell counts differ between Sjögren’s disease and non-Sjögren sicca and associate with key disease features.

**What are the implications of the main findings?**

- Tuft cells likely contribute to salivary gland epithelial integrity and secretory homeostasis, supporting a broader sentinel/regulatory role in gland biology.
- Tuft cell abundance may serve as a candidate tissue biomarker of salivary gland dysfunction/injury and supports future mechanistic and translational studies in Sjögren’s disease.

**Abstract:**

Tuft cells (TCs) are rare chemosensory epithelial cells that regulate mucosal homeostasis in multiple organs, but their role in salivary gland (SG) biology remains poorly defined. This study aimed to define TC structure in mice submandibular glands (SMGs), determine how TC loss affects gland organization and function, and evaluate whether TC abundance in human minor SGs is associated with Sjögren’s disease (SjD) features. Specifically, TC ultrastructure and ductal localization were characterized in female and male C57BL/6J mouse SMGs by transmission electron microscopy and immunostaining. Wild-type and C57BL/6J-*Pou2f3^-/-^* (TC-deficient mouse strain) SMGs were analyzed by histology and bulk RNA-seq, and salivary function was assessed by saliva flow and proteomics. Human minor SG biopsies from SjD and non-Sjögren sicca (nSjD) patients were analyzed by immunostaining and Poisson regression. In mice SMGs, TCs showed conserved ultrastructural features and localization in both sexes. TC loss was associated with marked sex-biased transcriptome remodeling, morphological disruption, and altered saliva quantity and quality. In humans, TC counts differed between nSjD and SjD groups and were associated with salivary flow, serologic status, and histopathologic features. These findings support a role for TCs in SG epithelial integrity and suggest TC abundance as a candidate biomarker of SG dysfunction.

## 1. Introduction

Tuft cells (TCs) are chemosensory cells identified approximately sixty years ago in mouse and human epithelia by their unique bottle shape together with apical microvilli-rich tuft-like, dense actin filaments and deep-reaching rootlets [1,2,3,4,5]. However, a lack of genetic tools and specific biomarkers limited the understanding of their role at that time, until they were shown to modulate inflammatory responses in the intestinal epithelium in 2016 [6,7,8]. Subsequently, several other groups have shown that TCs not only modulate inflammation, but also are directly involved in development, regeneration and carcinogenesis processes in several other organs [9,10,11,12,13,14]. Regarding TC identity, POU2F3 (POU class 2 homeobox 3) is the first identified master regulator transcription factor required for the generation of TCs and mice lacking this transcription factor lack TCs across all organs [6,13,15,16]. Additionally, *Pou2f3* deficiency does not impact on global immunity [6] and, therefore, previous studies have validated *Pou2f3^-/-^* mice as a suitable model to study the function of TCs [6,17,18,19]. Functionally, TCs express canonical chemosensory signaling components (e.g., TasR, Gnat3 and Trpm5) similar to type II taste cells as well as effector molecules such as acetylcholine, eicosanoid, and interleukin (IL)-25 [6,7,8,20,21,22,23,24,25,26,27], which collectively enable TCs to detect microbial and other luminal stimuli and signal to neighboring cells [28,29,30]. Despite these advances in understanding TC biology, additional structural and functional studies on TCs are warranted, as the role of these cells in health and disease remains poorly understood as well as their potential use as therapeutic targets.

Until only recently TC studies in salivary glands (SGs) were restricted to rats [3,31,32]. In this species, ultrastructural analysis indicated that TCs are located within SG ducts and share fine details with their counterparts in different organs, such as distinct apical microvilli and tubulovesicular system [33,34,35,36]. However, these initial rat studies were not subsequently extended to other species. This motivated our recent studies, which identified TCs in mouse and human submandibular glands (SMGs) [37,38]. Additionally, TCs have been detected in benign (i.e., Warthin tumor and pleomorphic adenoma) and malignant (i.e., adenoid cystic carcinoma) SG neoplasms, which indicates that these cells may play a role in SG disease [39,40]. Nonetheless, despite recent efforts to understand TC biology in SGs, substantial gaps remain in our understanding of their structure and function within these organs.

Here, we compare the ultrastructure and distribution of TCs in male and female mouse SMGs. Then, we demonstrate differences in the SMG transcriptome, tissue architecture and saliva proteomic profile of mice lacking TCs. Finally, we extend our investigation to human minor SG biopsies from individuals with sicca symptoms classified as either primary Sjögren’s disease (SjD; 2016 ACR/EULAR criteria) or non-Sjögren sicca (nSjD) [41]. Collectively, this study expands our understanding of TC biology in mouse and human SGs and serves as a basis for future studies to define the specific signaling mechanisms by which TCs regulate salivary tissue homeostasis.

## 2. Materials and Methods

### 2.1. Animals

Female and male C57BL/6J and C57BL/6J-*Pou2f3^em1Cbwi^*/J (i.e., C57BL/6J-*Pou2f3^-/-^*) mice from Jackson Laboratory at 8 weeks of age were euthanized with CO_2_ (30% per minute) followed by abdominal exsanguination and removal of the SMGs. All animal management and procedures were approved by the University at Buffalo’s Institutional Animal Care and Use Committee Approval Number: PROTO202400054.

### 2.2. Human Minor Salivary Glands

Minor SGs from forty adult individuals presenting with complaints of dry mouth and/or dry eyes who met the 2016 classification criteria for SjD, as defined by the American College of Rheumatology and the European League Against Rheumatism (ACR/EULAR), were included in the SjD group [41,42]. The control group (nSjD group) consisted of minor SGs from twenty individuals with sicca symptoms (including xerostomia and/or xerophthalmia) who did not fulfill the 2016 ACR/EULAR classification criteria for SjD after standardized evaluation [42,43,44]. Specifically, these individuals did not reach the minimum classification score of 4 required for SjD. The reasons for not fulfilling the criteria varied among patients but necessarily included the absence of anti-SSA/Ro antibodies and/or a focus score < 1 on minor salivary gland biopsy, with focus score defined as the number of lymphocytic foci, each composed of ≥50 mononuclear cells, per 4 mm^2^ of glandular tissue [45,46]. Additionally, some patients also presented unstimulated salivary flow rate > 0.1 mL/min, a negative Schirmer’s test, and/or a negative Ocular Staining Score. Moreover, although historically regarded as a benign and idiopathic condition, nSjD is now recognized as a distinct and underexplored clinical entity associated with a high symptomatic burden (e.g., persistent oral dryness, fatigue, oral discomfort, and functional and psychosocial impairment) [47]. Exclusion criteria were the same for both groups and were based on ACR/EULAR classification criteria, including pregnancy, prior use of anticholinergic drugs, history of head and neck radiotherapy, HIV/AIDS, hepatitis C infection, sarcoidosis, amyloidosis, neoplasms, graft-versus-host disease, and IgG4-related disease. Moreover, clinical variables collected included: demographic characteristics, symptom duration, medication use, serological status for anti-Ro/SSA and anti-La/SSB antibodies, and ocular test results (Schirmer test and Ocular Staining Score [OSS]). Also, symptoms of dryness (i.e., xerostomia/hyposalivation and xerophthalmia) were assessed in conjunction with the Clinical Oral Dryness Score (CODS) [48]. Moreover, unstimulated saliva was collected using the spitting method over a 10 min period, as previously described [49]. All samples were collected in the morning under fasting conditions and were supervised by trained examiners. Salivary flow rate was expressed in mL/min. Participants were consecutively recruited between 2022 and 2024. Finally, analyses of these specimens were conducted under the guidelines and with the approval of the Federal University of Minas Gerais Institutional Research Ethics Committee (No. 60804622.9.0000.5149), with informed consent obtained for each patient.

### 2.3. Hematoxylin and Eosin Staining

Mouse SMGs were collected, washed with PBS (PBS; Corning Inc., Corning, NY, USA) and fixed in formalin (Thermo Fisher Scientific, Waltham, MA, USA) for one day. After dehydration with ethanol, tissue blocks were created using paraffin wax (Leica Biosystems, Buffalo Grove, IL, USA), and 5-μm-thick tissue sections were obtained. Next, we performed H&E staining (Abcam, Cambridge, UK) to investigate morphological changes [50]. Specifically, SMG tissue sections from each group were deparaffinized with xylene (Sigma-Aldrich, St. Louis, MO, USA) and rehydrated with serial ethanol solutions (100%, 70%, and 50%; Sigma-Aldrich, St. Louis, MO, USA) followed by distilled water. Later, the rehydrated sections were stained with Harris Hematoxylin for 6 min. Then, hematoxylin-stained sections were washed with distilled water for 2 min, treated with 0.5% (*w*/*v*) lithium carbonate (Li_2_CO_3_) solution (Sigma-Aldrich, St. Louis, MO, USA) for 1 min, and rinsed with distilled water for 1 min. Following, slides were washed with 95% ethanol for 1 min, followed by a 1 min incubation with Eosin and washed with 95% ethanol for 1 min. Subsequently, sections were washed three times with 100% ethanol, cleared in xylene, and mounted with a xylene-based mounting medium (Thermo Fisher Scientific, Waltham, MA, USA). Samples were examined using the Nikon NiE Upright microscope (Nikon Instruments Inc., Melville, NY, USA). In mouse SMGs, H&E-stained sections were examined qualitatively to compare glandular architecture, acinar and ductal morphology and inflammatory cell infiltrates between experimental groups. For human minor SG samples, histological parameters—including focus score calculation, grading of atrophy, fibrosis, and ductal dilatation, and assessment of germinal center–like structures and lymphoepithelial lesions—were evaluated using established criteria for salivary gland histopathology in primary SjD [41]. Finally, the extent of adipose tissue replacement and acinar dilatation was also qualitatively graded as absent, mild, moderate, or severe.

### 2.4. Transmission Electron Microscopy

Mouse SMG were fixed using 2% (*v*/*v*) glutaraldehyde and 2% (*v*/*v*) formaldehyde (prepared from paraformaldehyde) in 0.1 M cacodylate buffer, pH 7.2, overnight at 4 °C. Then, fixed specimens were then rinsed four times for 10 min each with 30 mM HEPES, pH 7.2, 70 mM NaCl, and 6% (*w*/*v*) sucrose, rinsed three times with 20 mM Tris, pH 7.2, containing 120 mM NaCl and 5 mM CaCl_2_, postfixed with osmium tetroxide 1% (*w*/*v*) OsO_4_, 70 mM NaCl, 5 mM CaCl_2_, 30 mM HEPES buffer, pH 7.4, for 10 min, and rinsed three times for 10 min each with distilled water. Next, fixed samples were stained overnight in aqueous 0.5% (*w*/*v*) uranyl acetate, pH 6.0, at room temperature and infiltrated with Epon-Araldite epoxy resin (Electron Microscopy Sciences, Hatfield, PA, USA). The infiltrate was placed in fresh resin in ballistic electron-emission microscopy (BEEM) embedding capsules (Electron Microscopy Sciences, Hatfield, PA, USA) and polymerized at 60 °C. Later, sections of 70 nm thickness were cut on a Leica ultra-cut (UCT) microtome (Leica Microsystems, Buffalo Grove, IL, USA) and stained with 5% (*w*/*v*) uranyl acetate and Sato’s triple lead salt stain consisting of 1% (*w*/*v*) lead citrate, 1% (*w*/*v*) lead acetate, 1% (*w*/*v*) lead nitrate, and 2% (*w*/*v*) sodium citrate. Finally, samples were viewed and photographed with a Japan Electro Optics Laboratories (JEOL 1400) transmission electron microscope (JEOL USA Inc., Peabody, MA, USA).

### 2.5. Bulk RNA-Sequencing: RNA Extraction, Library Preparation, Sequencing, Differentially Expressed Gene (DEG) and Enrichment Analyses

Submandibular gland (SMG) tissue was collected from each mouse, snap-frozen and stored at −80 °C, and processed for bulk RNA-sequencing by the University at Buffalo Genomics Core. Total RNA was extracted from SMG tissue using the Qiagen RNeasy Micro Kit (Qiagen, Hilden, Germany) according to the manufacturer’s instructions. RNA quantity and quality were assessed using Qubit (Invitrogen, Waltham, MA, USA) and an Agilent Fragment Analyzer (Agilent Technologies, Santa Clara, CA, USA), respectively. A quantity of 100 ng of total RNA per sample was used to generate stranded, ribosomal RNA–depleted libraries using the Illumina RiboZero Total Stranded RNA library preparation kit (Illumina, San Diego, CA, USA). Following library preparation and quality control, libraries were pooled to 10 nM, and the pool concentration was determined using the QuantaBio Universal qPCR reaction kit (QuantaBio, Beverly, MA, USA). After dilution and denaturation, pooled libraries were sequenced on an Illumina NovaSeq 6000 system (Illumina, San Diego, CA, USA) using paired-end 100 bp reads (2 × 100; PE100). Next, raw read quality was assessed using standard sequencing QC procedures implemented by the Genomics Core and/or downstream computational pipeline. For computational analysis, transcript abundance was quantified using Salmon (v1.10.1) against the Gencode M35 reference transcriptome (GRCm39/mm39). Differential expression analysis was performed with DESeq2 [51], and genes were considered differentially expressed using a Benjamini–Hochberg adjusted *p*-value (FDR) < 0.05. Volcano plots and heatmaps were generated in R (v4.5.2) using ggplot2 (4.0.2) and ComplexHeatmap (2.26.1), respectively [52,53]. For enrichment analyses, gene sets were defined using FDR < 0.05 and |log2 fold change| > 1, and were analyzed using the Core Analysis function in Qiagen Ingenuity Pathway Analysis (IPA, v9.0) [54]. Finally, RNA-seq datasets have been deposited in the Gene Expression Omnibus (GEO) under accession GSE320144.

### 2.6. Immunofluorescence

Mouse SMG sections were blocked for 90 min at room temperature in blocking buffer containing 0.5% Triton X-100 (Sigma-Aldrich, St. Louis, MO, USA), 1% bovine serum albumin (BSA, Sigma-Aldrich, St. Louis, MO, USA), and 5% normal goat serum (NGS, Cell Signaling Technology, Danvers, MA, USA), then incubated overnight at 4 °C with primary antibodies diluted in blocking buffer: anti-POU2F3 (HPA019652; 1:1000; Sigma-Aldrich, St. Louis, MO, USA) and keratin 8 (KRT8) hybridoma supernatant (TROMA-I-s, 17 µg/mL; 1:16; Developmental Studies Hybridoma Bank, University of Iowa, Iowa City, IA, USA). Sections were washed four times with wash buffer (0.1% Triton X-100 in PBS) and incubated for 1 h at room temperature with secondary antibodies diluted in blocking buffer: goat anti-rabbit IgG Alexa Fluor 488 (ab150081; 1:500; Abcam, Cambridge, UK) and goat anti-rat IgG Alexa Fluor 647 (ab150159; 1:500; Abcam, Cambridge, UK). Sections were then washed four times with PBS containing Tween-20 and mounted using an aqueous mounting medium with DAPI (Fluoroshield, ab104139-1002; Abcam, Cambridge, UK). Finally, sections were imaged using the Andor Dragonfly Confocal Microscope (Andor Technology Ltd., Belfast, UK).

### 2.7. Immunohistochemistry

Five μm thick minor human SG serial sections were obtained using a microtome and mounted on polarized slides (EasyPath^®^, Indaiatuba, SP, Brazil). After deparaffinization, rehydration, and antigen retrieval, we used the Trilogy™ solution (Cell Marque^®^, a Sigma-Aldrich Company, Rocklin, CA, USA). The staining procedure was performed using the EnVision FLEX Mini Kit, high pH (Agilent Dako^®^, Santa Clara, CA, USA). POU2F3 immunostaining was performed with a monoclonal anti-POU2F3 antibody (dilution 1:100; antibody incubation for 2 h; Cell Signaling Technology, Danvers, MA, USA), with human intestinal tissue used as a positive control. Finally, immunostaining was evaluated by a single examiner by manual counting of all POU2F3^+^ cells in each microscopic field at 400× magnification, with each field corresponding to an area of 0.1024 mm^2^.

Data normality was appraised using the Shapiro–Wilk and Kolmogorov–Smirnov tests, which indicated non-normal distributions. Categorical variables were compared using the χ^2^ test or Fisher’s exact test, as appropriate. For comparisons between two groups, the Mann–Whitney U test was used. A Poisson regression model with a log-linear link function was constructed to evaluate the association between independent variables and the count of tuft cells. Salivary gland area (mm^2^) was included as a covariate, and all independent variables, encompassing clinical, serological, and histopathological features, were adjusted for this parameter. The results are expressed as rate ratio (RR) along with their respective 95% confidence intervals (CI). Finally, all analyses were performed using R programming language (version 4.4.1; R Core Team, 2025).

### 2.8. Saliva Collection

Mice were anesthetized intraperitoneally with an xylazine (5 mg/kg) and ketamine (80 mg/kg) solution followed by intraperitoneal injection with pilocarpine (2.5 mg/kg in PBS, (Sigma-Aldrich, St. Louis, MO, USA)) to stimulate saliva secretion. Saliva was then collected for 20 min and the volume of the collected saliva was measured and calculated using the following equation, as described previously [55]:

$$\mathrm{Saliva flow rate}=\frac{\text{Stimulated saliva flow rate }(\mathrm{\mu L})}{\mathrm{Mouse body weight}(g)\times \mathrm{collection time}(\min)}$$

Statistical significance in saliva volume was assessed using unpaired two-tailed Student’s *t*-tests, comparing C57BL/6J and C57BL/6J-*Pou2f3^-/-^* mice within each sex (male WT vs. male knockout; female WT vs. female knockout). A *p* value ≤ 0.05 was considered statistically significant. Analyses were performed using GraphPad Prism (version 8.4.2). Collected saliva was then subjected to liquid chromatography–tandem mass spectrometry (LC–MS/MS), as detailed below.

#### 2.8.1. Salivary Protein Digestion

Protein concentrations of saliva samples were determined using the bicinchoninic acid (BCA) assay (Thermo Fisher Scientific, Waltham, MA, USA). Thirty micrograms of total protein from each sample were prepared for digestion, and the volume was adjusted to 60 µL with 0.5% SDS buffer. Proteins were reduced with 10 mM dithiothreitol at 56 °C for 30 min and alkylated with 20 mM iodoacetamide at 37 °C for 30 min in the dark. Both reduction and alkylation steps were carried out with constant vortexing in a Thermomixer at 550 rpm. Protein precipitation was performed by adding 1 and 5 volumes of chilled acetone followed by vortexing, and the mixture was incubated at −20 °C for 3 h. After centrifugation at 20,000× *g* for 30 min at 4 °C, the supernatant was discarded, and the protein pellets were washed with 200 µL methanol. The protein pellets were reconstituted in 50 mM Tris-FA. Trypsin (Sigma-Aldrich, St. Louis, MO, USA), activated with 50 mM Tris-FA, was added to the protein pellets at a protein to trypsin ratio of 20:1, and tryptic digestion was carried out at 37 °C overnight with constant vortexing. Digestion was terminated by adding 1% FA. Finally, samples were centrifuged at 20,000× *g* for 30 min at 4 °C, and the supernatants were carefully transferred to LC vials.

#### 2.8.2. Salivary LC-MS Analysis

Liquid chromatography–tandem mass spectrometry (LC-MS/MS) was conducted using a trapping nano-flow LC-Orbitrap Astral MS system, which comprised a Dionex Ultimate 3000 nano LC system, a Dionex Ultimate 3000 gradient micro-LC system with a WPS-3000 autosampler, and an Orbitrap Astral mass spectrometer (Thermo Fisher Scientific, Waltham, MA, USA).

A single injection of 250 ng derived peptides was analyzed for each sample. A trapping column (100 µm ID × 5 cm, packed with 5 µm C18, CoAnn Technologies, Richland, WA, USA) was employed prior to nano LC column (75 μm ID × 30 cm, packed with 1.7 μm C18, CoAnn Technologies, Richland, WA, USA) separation for matrix component removal and selective peptide delivery. Mobile phases A and B were 0.1% formic acid (FA) in 2% acetonitrile, and 0.1% FA in 88% acetonitrile, respectively. The LC gradient profile was as follows: 4–37% B for 39 min; 37% to 97% B for 0.1 min; and isocratic at 97% B for 3 min.

Mass spectrometry operated in data-independent acquisition (DIA) mode. MS1 spectra were acquired in the *m*/*z* range of 380 to 980 at a resolution of 180k. The normalized Automatic Gain Control (AGC) target for MS1 was set at 500%, with a maximum injection time of 5 ms. Precursor ions were isolated using a 2 Th-wide window and fragmented by higher-energy collision-induced dissociation (HCD) at a normalized collision energy of 25%. MS2 spectra were acquired in the Astral (AST) mass analyzer. The normalized Automatic Gain Control (AGC) target was set at 200%, with a maximum injection time of 3 ms.

#### 2.8.3. Protein Identification and Quantification

The MS raw files were analyzed by searching against the Uniprot-SwissProt Mouse database (accessed January 2024, containing 17,156 protein entries) using DIA-NN (v1.8.1) in library-free mode. Trypsin was selected as the protease with one allowed missed cleavage. Search parameters included a maximum of three modifications, including cysteine carbamidomethylation, methionine oxidation and N-terminal acetylation. The precursor and protein FDR% was set to 1%. Mass accuracy was set to 3 ppm for MS1 and 7 ppm for MS2. The scan window was set to 12.

Differential abundance analysis was performed using limma (R v4.4.2) on normalized protein intensities from DIA-NN v1.8.1. Values were log_2_-transformed, and no additional normalization was applied prior to limma analysis. Proteins were required to be quantified in ≥70% of samples per group, and missing values were imputed using a group-aware K-nearest neighbor (KNN) method (k = 2). Comparisons were performed between female C57BL/6J vs. female C57BL/6J-*Pou2f3^-/-^* and male C57BL/6J vs. male C57BL/6J-*Pou2f3^-/-^*. Multiple testing correction was not applied due to the limited number of significantly changed proteins. Differentially expressed proteins were defined as *p* < 0.05 and |fold change| ≥ 1.4. Finally, heatmaps were generated from the top 50 proteins ranked by |log_2_FC|, using row-wise Z-score–scaled log_2_-transformed intensities, with hierarchical clustering based on Euclidean distance and complete linkage (pheatmap, R).

STRING protein–protein interaction networks were generated using the same top-50 protein lists in Mus musculus (STRING v12.0), with a minimum required interaction score of 0.400, using all available evidence sources (text mining, experiments, curated databases, co-expression, neighborhood, gene fusion, and co-occurrence). Disconnected nodes were not hidden, and no additional interactors were added (query proteins only). Finally, proteins not mapped to STRING were 2 (male) and 5 (female), respectively.

## 3. Results

### 3.1. Tuft Cell Ultrastructure and Distribution Are Similar in Female and Male Mouse Submandibular Glands

**Figure 1:**
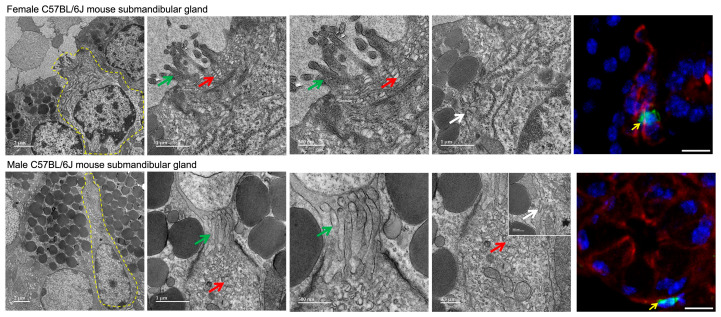
Fine structure of TC in 8 wk female and male C57BL/6J mouse SMGs. Shown are transmission electron micrographs of TCs in female and male C57BL/6J mice SMG at 8 weeks. The higher-magnification TEM panels show enlarged views of regions from the adjacent lower-magnification images. Yellow dotted lines indicate TCs. Green arrows denote characteristic apical microvilli, red arrows indicate apical vesicles and white arrows indicate lateral projections (i.e., cytospinules). Images are representative of two mice per group. POU2F3 (TC marker, green color) and KRT8 (ductal marker, red color) staining show TC (yellow arrows) in ducts. Images are representative of 3 mice per group. Scale bars = 10 µm.

To compare the ultrastructure of TCs within male and female SMGs, we evaluated their fine structure using transmission electron microscopy. In both sexes, TCs were identified in ducts by their long finger-like microvilli, numerous electron-lucent vesicles and tubules of varying sizes in the apical cytoplasm as well as lateral projections (i.e., cytospinules) on the plasma membrane (Figure 1). Moreover, immunofluorescence staining for POU2F3 (i.e., TC-defining transcription factor) and KRT8 (i.e., ductal marker) confirmed the presence and ductal localization of TCs in SMGs from both females and males (Figure 1). Together, these findings indicate that TC ultrastructural features and ductal distribution are similar in female and male mouse SMGs.

### 3.2. Tuft Cell Loss Is Associated with Sex-Specific Transcriptomic Alterations in Female and Male Submandibular Glands

**Figure 2:**
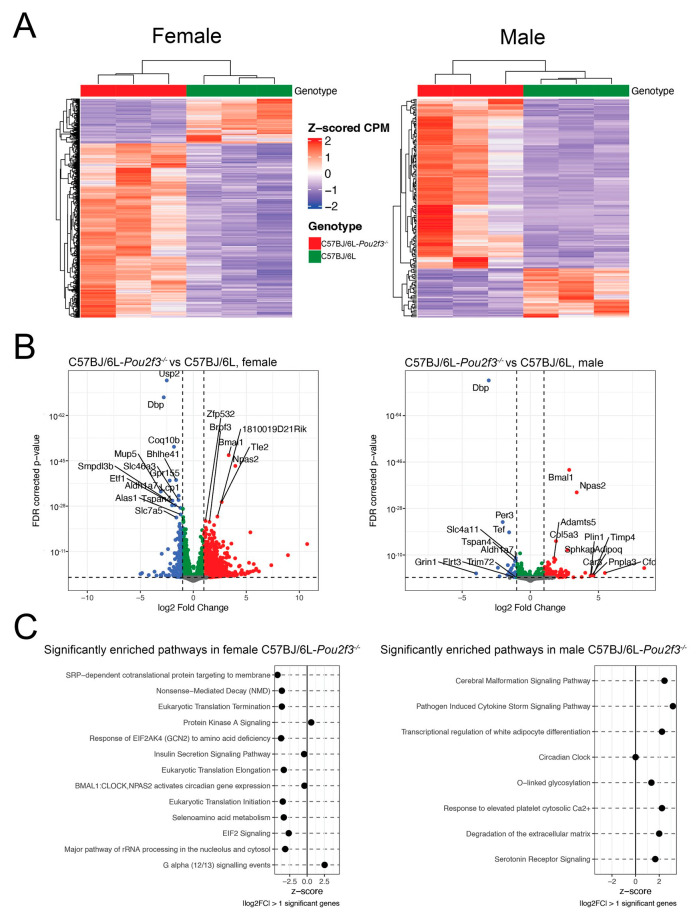
Bulk RNA-seq of SMG reveals sex-specific transcriptional reprogramming with TC loss. (**A**) Heatmaps of differentially expressed genes in female and male C57BL/6J and C57BL/6J-*Pou2f3*^-/-^ mouse SMGs at 8 wks (DESeq2; padj < 0.05, |log_2_FC| > 1). Values are Z-scored CPM per gene; rows/columns hierarchically clustered; column color bars indicate genotype (green indicates wild-type and red indicates C57BL/6J-*Pou2f3*^-/-^ mice). (**B**) Volcano plots indicate up-regulated (red), down-regulated (blue) and significant, but not strongly up- or down-regulated (green) genes in SMG C57BL/6J-*Pou2f3*^-/-^ mice, compared to wild-type counterpart. Multiple testing controlled by Benjamini–Hochberg FDR. (**C**) IPA activation z-scores for the same contrasts, with dot size reflecting gene counts; positive z-score indicates predicted activation, negative z-score inhibition. Input sets were defined by DESeq2 (padj < 0.05, |log_2_FC| > 1); background = all expressed genes. n = 3 per sex per genotype (total n = 12); multiple testing controlled by Benjamini–Hochberg.

To better define the global gene expression patterns, we performed bulk RNA-sequencing on 8-week-old SMGs from C57BL/6J and C57BL/6J-*Pou2f3*^-/-^ female and male mice. We utilized three biological replicates of SMGs from each group to capture biological variability and ensure robust downstream inferential analysis. Particularly, this method allowed us to investigate the altered gene expression pattern in the C57BL/6J-*Pou2f3*^-/-^ mice and how it compares to C57BL/6J control mice. Specifically, unsupervised hierarchical clustering of significantly differentially expressed genes (padj < 0.05 and |log_2_ fold-change| > 1) showed clear separation of samples by genotype in both sexes, indicating a robust *Pou2f3*-dependent transcriptional signature (Figure 2A). At a false discovery rate (FDR) of 0.05, our study identified a total of 1742 differentially expressed genes (DEGs) in the SMG from C57BL/6J-*Pou2f3*^-/-^ female mice, with 1386 genes being up-regulated and 356 genes showing down-regulation. In contrast, a much lower number of DEGs were identified in SMGs from C57BL/6J-*Pou2f3*^-/-^ male mice, totaling 175 genes, with 136 being up-regulated and 39 showing down-regulation. Together, these findings indicate that TC loss is associated with sex-specific transcriptional imbalance in SMG, which is notably greater in C57BL/6J-*Pou2f3*^-/-^ female mice (Figure 2B). Finally, to gain insights into the biological relevance of the global transcriptomic differences, we performed pathway analysis based on DEGs with padj < 0.05 and |log_2_ fold-change| > 1 on SMGs from C57BL/6J and C57BL/6J-*Pou2f3*^-/-^ female and male mice. As shown in Figure 2C, pathway analysis revealed a marked sex bias, where in SMG from C57BL/6J-*Pou2f3*^-/-^ female mice, pathways predominantly reflected down-regulation of translational/secretory and metabolic programs. In contrast, in SMGs from C57BL/6J-*Pou2f3*^-/-^ male mice, several up-regulated pathways included O-linked glycosylation, inflammatory and extracellular matrix-related signaling (Tables S1 and S2). Together, these findings indicate that TC loss is associated with sex-specific transcriptional remodeling of mouse SMG.

### 3.3. Tuft Cell Absence Impairs Submandibular Morphology

**Figure 3:**
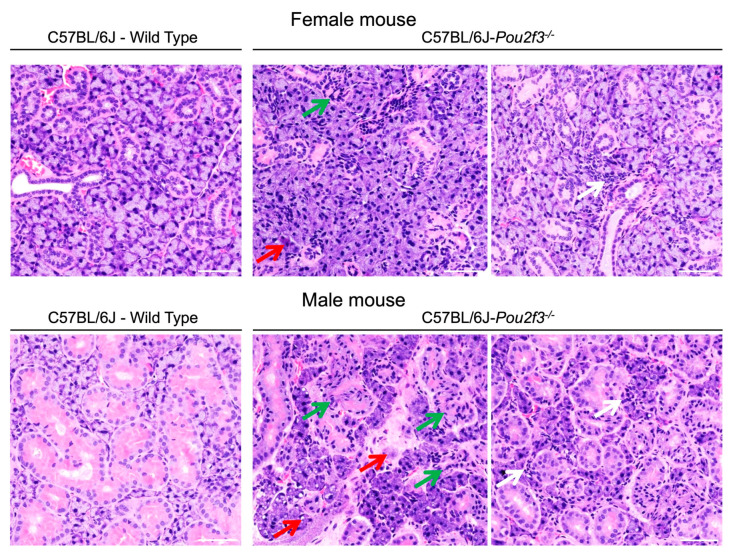
TC loss damages both female and male mouse SMG structure. SMGs from female and male C57BL/6J wild-type mouse SMG at 8 weeks display preserved acinar and ductal morphology. In contrast, SMG from C57BL/6J-*Pou2f3*^-/-^ mice exhibit disrupted architecture with ductal (green arrows) and acinar degeneration (red arrows) as evidenced by nuclear atypia, cell vacuolization or apoptosis. Also, C57BL/6J-*Pou2f3*^-/-^ mice displayed inflammatory cell infiltrate aggregates (white arrows). Images are representative of three mice per group, ≥5 fields per gland were examined in a blinded fashion. Scale bars = 100 µm.

Having defined sex-specific transcriptomic signatures of tissue remodeling and inflammation in the SMG of C57BL/6J-*Pou2f3*^-/-^ female and male mice, we next examined gland architecture by routine histology. As shown in Figure 3, histological analysis indicated that SMGs from both female and male C57BL/6J-*Pou2f3*^-/-^ mice showed marked structural abnormalities compared with wild-type controls, including ductal and acinar disorganization as well as the presence of interstitial inflammatory cell aggregates. These changes were absent in wild-type mice. Together, these observations indicate that TC loss is associated with architectural disruption of SMGs in both female and male mouse SMGs.

### 3.4. Tuft Cell Loss Alters Saliva Quantity and Quality

**Figure 4:**
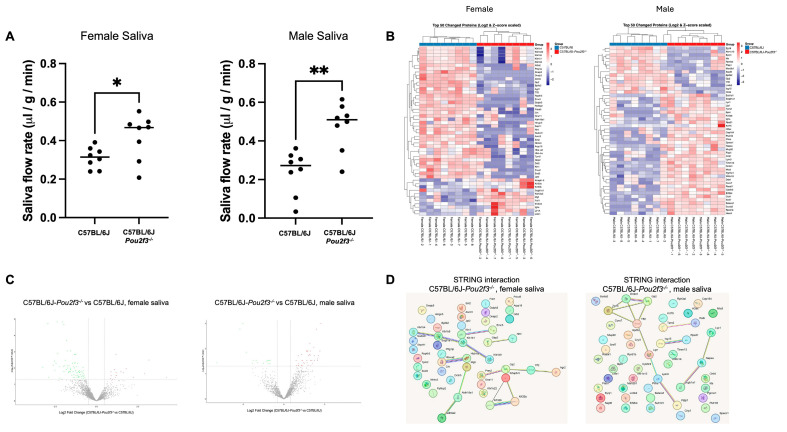
TC loss increases salivary output and remodels the saliva proteome in a sex-divergent manner. (**A**) Saliva was collected for 20 min from C57BL/6J and C57BL/6J-*Pou2f3*^-/-^ mice at 8 wks after pilocarpine administration (2.5 mg/kg, i.p.). Volumes were normalized to body weight and collection time. Statistics: two-tailed unpaired *t*-test; * *p* < 0.05 and ** *p* < 0.01. (**B**) Proteomic profiling of collected saliva by label-free mass spectrometry revealed extensive sex-specific remodeling. Heatmaps show the top 50 differentially abundant salivary proteins for females (left) and males (right), ranked by |log_2_FC| (limma). Columns represent individual mice, rows represent UniProt protein IDs; values are row-wise Z-scores of log_2_ intensities. Hierarchical clustering separates genotypes within each sex, indicating robust, sex-dependent shifts in saliva composition. (**C**) Volcano plots revealed genotype-dependent proteomic remodeling: in saliva from C57BL/6J-*Pou2f3*^-/-^ female mice, 17 proteins were significantly up-regulated and 75 down-regulated; in saliva from C57BL/6J-*Pou2f3*^-/-^ male mice, 39 proteins were up-regulated and 16 down-regulated (significance: nominal *p* < 0.05 and |fold change| ≥ 1.4). (**D**) STRING protein–protein interaction networks for 50 proteins in female and male C57BL/6J-Pou2f3^-/-^ saliva. n = 8 biological replicates per sex per genotype (total = 32).

**Figure A1:**
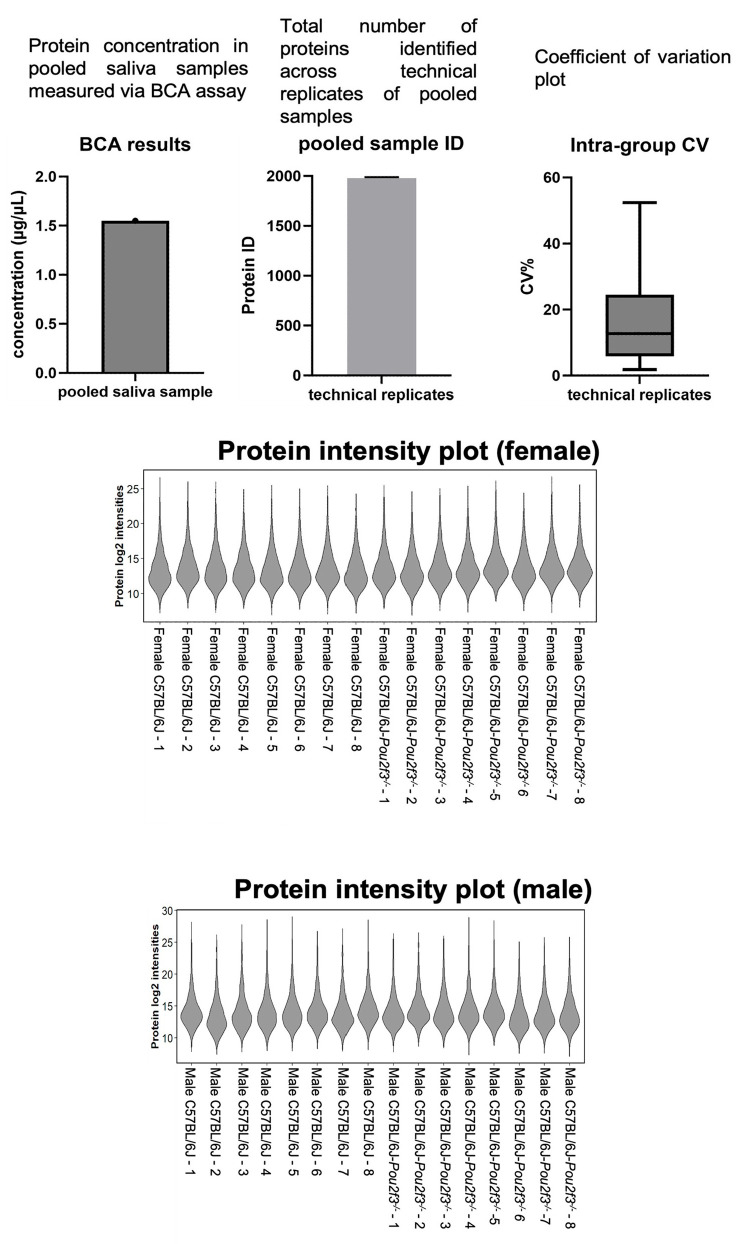
Quality control and normalization of label-free saliva proteomics in C57BL/6J (WT) and C57BL/6J-*Pou2f3^-/-^* saliva. Pooled saliva yielded ~1.5 µg/µL protein by BCA assay. Then, technical replicates identified ~2000 proteins. Also, coefficient of variation (CV) analysis showed a 13% median, confirming reproducible quantification. Finally, violin plots of protein intensity distributions demonstrated comparable normalization across wild-type and C57BL/6J-*Pou2f3^-/-^*. n = 8 biological replicates per sex per genotype.

Next, we investigated the impact of TC loss on SG function by analyzing saliva quantity and quality from wild-type and C57BL/6J-*Pou2f3*^-/-^ mice. Specifically, female and male C57BL/6J-*Pou2f3*^-/-^ mice secreted significantly more saliva than their wild-type counterparts, revealing a hypersecretion phenotype (Figure 4A). Then, saliva sample validation for proteomic analysis indicated adequate protein yield, broad coverage and reproducibility (Figure A1). Remarkably, our results demonstrate that unsupervised hierarchical clustering of the top 50 differentially abundant proteins for each sex distinctly separated C57BL/6J from C57BL/6J-*Pou2f3*^-/-^ saliva samples, indicating a robust genotype-driven salivary proteomic signature (Figure 4B). Moreover, differential abundance analysis (limma; nominal *p* < 0.05 and |fold change| ≥ 1.4; see Methods) revealed sex-divergent remodeling of saliva composition, where female C57BL/6J-*Pou2f3*^-/-^ saliva displayed 17 up-regulated and 75 down-regulated proteins compared to C57BL/6J, whereas male C57BL/6J-*Pou2f3*^-/-^ saliva showed the opposite pattern with 39 up-regulated and 16 down-regulated proteins compared to C57BL/6J (Figure 4C). Together, these data establish that TC loss is associated with saliva hypersecretion and sex-divergent proteomic remodeling.

Finally, to assess whether differentially abundant proteins in saliva from C57BL/6J-*Pou2f3*^-/-^ female mice represented functionally connected modules, the 50 most significantly altered proteins (including both increased and decreased proteins) were subjected to STRING protein–protein interaction analysis. As shown in Figure 4D, network analysis of the proteins revealed two dominant interaction modules: a protease-centered cluster enriched for kallikrein family members (e.g., Klk1b1, Klk1b4 and Klk1b9) and an epithelial structural cluster containing keratin/keratin-associated proteins (e.g., Krt33a, Krt33b, Krtap6-5). Furthermore, smaller paired clusters were observed, including secretory-associated chain (i.e., Prss2, Gp2, Tff2, and Agr2), hemoglobin subunits (i.e., Hba-a2 and Hbb-bs) and an interferon-associated pair (i.e., Gbp2 and Nmi), along with several additional proteins that remained weakly connected or isolated under the applied STRING parameters. In contrast, in C57BL/6J-*Pou2f3*^-/-^ male saliva we observed a dominant secretory/mucosal module (Dmbt1, Gp2, Tff2, and Zg16) that is functionally connected to a lipid-handling cluster including Lipf, Lpin3, and Plpp1. Smaller connected groups were also observed, including an antimicrobial/epithelial-associated chain linking Lyz1, Napsa, and Scgb1a1, whereas several remaining proteins were weakly connected or isolated under the applied STRING parameters. Together, these STRING networks suggest that TC loss reshapes the salivary proteome into sex-specific and coordinated protein modules.

### 3.5. Tuft Cell Numbers Are Altered and Associate with Disease Features in Sjögren’s

**Figure 5:**
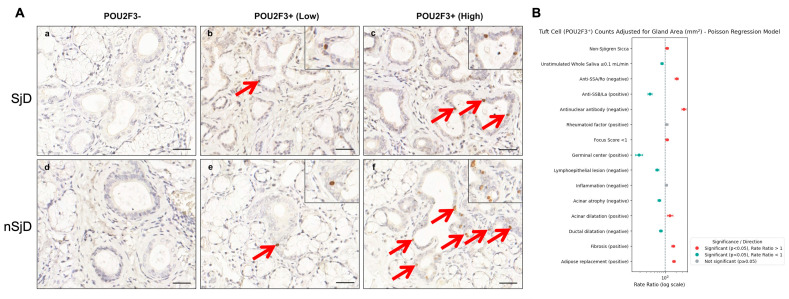
Tuft cells are heterogeneous and associate with Sjögren’s features. (**A**) Representative minor SG biopsies (POU2F3 immunostaining) illustrating variable numbers of TCs (red arrows and insets) across SjD (**a**–**c**) and nSjD (**d**–**f**) groups. Cohort size: n = 20 nSjD and n = 40 SjD patients. Scale bars represent 50 µm. (**B**) Forest plot from the Poisson regression model for the number of TCs (POU2F3+) adjusted for salivary gland area. Dots represent rate ratios, and horizontal bars denote 95% confidence intervals. Values to the right of the dashed vertical line (Rate Ratios > 1) indicate a positive association with the number of TC, whereas values to the left (Rate Ratios < 1) indicate a negative association. Variables on the y-axis are shown in abbreviated form, and the reference category for each variable (comparison factor in the model) is indicated in parentheses, as follows: Sicca, Non-Sjögren Sicca (vs. Sjögren); UWS < 0.1, Unstimulated Whole Saliva < 0.1 mL/min (vs. >0.1 mL/min); SSA/Ro–, Anti-SSA/Ro negative (vs. positive); SSB/La+, Anti-SSB/La positive (vs. negative); ANA–, antinuclear antibody negative (vs. positive); RF+, rheumatoid factor positive (vs. negative); FS < 1, focus score < 1 (vs. >1); GC+, germinal centers present (vs. absent); LEL–, lymphoepithelial lesion absent (vs. present); Inflammation–, inflammation absent (vs. present); Acinar atrophy–, acinar atrophy absent (vs. present); Acinar dilatation+, acinar dilatation present (vs. absent); Ductal dilatation–, ductal dilatation absent (vs. present); Fibrosis+, fibrosis present (vs. absent); Adipose replacement+, adipose replacement present (vs. absent).

Previous studies demonstrated that the number of TCs is altered in inflammatory diseases of the human intestine and airway epithelium [56,57]. Therefore, we investigated whether their abundance is altered in SjD, a systemic autoimmune disorder that damages salivary and lacrimal glands, leading to chronic sicca symptoms (hyposalivation and keratoconjunctivitis sicca) [58,59,60,61]. For this purpose, we studied two groups: patients with SjD defined by the 2016 ACR/EULAR classification criteria [42] and nSjD patients with sicca symptoms (e.g., dry mouth and dry eyes) who did not fulfill these criteria. Overall demographic, clinical, and histopathological features are summarized in Table S3, with Figure S1 highlighting the marked difference in focus score distribution between groups: the SjD group had a mean, median, and range of 3.03, 2.90, and 1.06–7.06, respectively, whereas the nSjD group had corresponding values of 0.48, 0.60, and 0.00–0.94. Then, we quantified TCs in minor SGs from these two groups and performed Poisson regression analysis to examine how TC counts per gland area were associated with clinical, serologic and histopathologic variables. As shown in Figure 5A and Table 1, TC numbers varied within and between groups. Notably, there were patients from both groups lacking TCs. Also, Figure 5B and Table A1 show how TC numbers associate with SjD clinico-serological and histologic features. Specifically, nSjD glands contained more TCs than SjD glands (RR = 1.12, 95% CI 1.02–1.23; *p* < 0.001). Additionally, reduced unstimulated whole salivary flow (≤0.1 mL·min^−1^) was associated with fewer TCs (RR = 0.82, 95% CI 0.75–0.89; *p* = 0.005). Serologically, anti-SSA/Ro-negative and ANA-negative patients had markedly higher TC counts (RR = 2.07, 95% CI 1.85–2.32 and RR = 3.27, 95% CI 2.86–3.73, respectively; both *p* < 0.001), whereas anti-SSB/La positivity was associated with substantially fewer TCs (RR = 0.38, 95% CI 0.33–0.44; *p* < 0.001). Moreover, rheumatoid factor positivity was not significantly associated with TC number (RR = 1.10, 95% CI 0.99–1.21; *p* = 0.079). As for histopathological features, a focus score <1 was associated with slightly higher TC counts (RR = 1.12, 95% CI 1.02–1.23; *p* = 0.018), while the presence of germinal centers corresponded to a pronounced reduction in TC numbers (RR = 0.19, 95% CI 0.15–0.24; *p* < 0.001). Additionally, glands without lymphoepithelial lesions or acinar atrophy had fewer TCs than those in which these lesions were present (RR = 0.60, 95% CI 0.54–0.67 and RR = 0.69, 95% CI 0.62–0.76, respectively; both *p* < 0.001). In contrast, the presence of acinar dilatation, fibrosis and adipose replacement was associated with higher TC counts (RR = 1.35, 1.66, and 1.73, respectively; all *p* ≤ 0.001), whereas absence of ductal dilatation was linked to fewer TCs (RR = 0.76, 95% CI 0.69–0.83; *p* < 0.001). The overall explanatory power (pseudo-R^2^) of the models ranged from 0.38 to 0.47. Together, these findings indicate that TC counts differed between the nSjD and SjD groups and were associated with key clinical, serologic, and histopathologic features relevant to SjD.

## 4. Discussion

TCs are rare chemosensory epithelial cells well characterized in several epithelia (e.g., intestine and airways), but their relevance in SGs has remained unclear. First, we define the ultrastructural features of TCs in female and male submandibular glands. Next, by integrating transcriptomic, morphologic and functional assays, we show that TC loss is associated with altered key signaling pathways, tissue architecture and secretory function in mouse SMGs. Finally, in minor human SGs, TCs are rare, and their abundance differs between SjD and nSjD groups and associates with clinical, serologic and histopathologic parameters across the SjD spectrum. Together, these multi-layered datasets support the fact that TCs are linked to SG integrity and dysfunction in both mouse and human species.

Our results indicate that TCs are rare ductal cells in both female and male mouse SMG and show fine structure similar to their counterparts in other organs, including apical microvilli, a prominent tubulo-vesicular system and lateral plasma-membrane projections (i.e., cytospinules) [33,62,63,64,65,66]. Notably, TCs are difficult to detect by TEM because they are rare; their shape varies with the plane of section and their diagonal orientation within the epithelium hinders visualization of the entire cell in a single micrograph [63,67], which likely contributes to their under-recognition in both mouse and human SG ultrastructural studies. Together, these findings confirm the presence of TCs in both female and male mouse SMGs.

Bulk RNA-seq revealed that TC loss is associated with sex-divergent transcriptional reprogramming in mouse SMGs. Specifically, SMGs from C57BL/6J-*Pou2f3^-/-^* female mice exhibited thousands of differentially expressed genes, whereas male glands showed fewer, but largely non-overlapping changes compared with their respective wild-type controls. In SMGs from female C57BL/6J-*Pou2f3^-/-^*, pathway analysis highlighted inhibition of translation and ribosome-associated pathways together with stress-sensing modules such as EIF2 signaling, consistent with dysregulation of protein synthesis and activation of the integrated stress response. Furthermore, inhibition of the insulin secretion pathway suggests that TC loss may impact SMG hormonal signaling. Moreover, dysregulation in the protein kinase A signaling pathway suggests a direct impact in saliva composition, since this molecule is directly involved in the exocytosis of protein storage granules and release of protein into saliva [68]. Also, inhibition of the G alpha (12/13) signaling pathway, which is a regulator of actin cytoskeletal and tight junction assembly [69,70,71], could link TC loss to epithelial architecture disruption. Finally, inhibition of the ROBO receptor signaling pathway could also affect SMG organization since this network is a key regulator of axon guidance [72] and epithelial branching morphogenesis [73,74]. In contrast, in SMGs from C57BL/6J-*Pou2f3^-/-^* male mice, the dominant transcriptional changes involved inflammatory, coagulation, O-linked glycosylation, and extracellular-matrix remodeling pathways and neuroendocrine signaling modules. In particular, activation of granulocyte adhesion, cytokine-storm and fibrin-clot signatures indicates heightened pro-inflammatory, pro-thrombotic and remodeling programs [75,76,77,78], while activation of serotonin receptor signaling pathways indicates altered serotonin-related signaling, a pathway previously shown to modulate salivary flow and protein content in rodent SGs [79,80,81]. Furthermore, enrichment of extracellular matrix degradation and altered cell adhesion pathways suggests that TC loss impacts on tissue remodeling and epithelial integrity. Finally, activation of the O-linked glycosylation pathway suggests increased mucin-type O-glycosylation that could alter saliva viscosity and lubrication [82]. Together, these findings indicate that TC loss is associated with sex-specific dysregulation in mouse SMGs.

To place these pathway-level changes in a physiologic context, we next examined representative DEGs with established roles in SG epithelial function (Table S4). In female C57BL/6J-*Pou2f3^-/-^* SMGs, the down-regulation of aquaporin water channels [83] (e.g., *Aqp5*), secretory trafficking components (e.g., *Sar1b* and *Arf4*), salivary-associated proteins [84,85] (e.g., *Pip*), and a broad cluster of ribosomal subunits and translation factors (e.g., *Rpl5* and *Rps14*) is consistent with impaired acinar secretory differentiation and biosynthetic capacity. This conclusion is further supported by the ectopic up-regulation of *Muc2*, a mucin more classically associated with intestinal goblet cells [86]. In male C57BL/6J-*Pou2f3^-/-^* SMGs, TC loss was associated with down-regulation of ion transport genes linked to ductal fluid and electrolyte secretion [87,88], including *Slc4a11* and *Ano4*, together with reduced expression of *Muc20*, a mucin that is detected in mouse SMGs [89]. Notably, these transcriptional changes in female and male C57BL/6J-*Pou2f3^-/-^* SMGs occurred despite saliva hypersecretion, raising the possibility that increased saliva output reflects abnormal fluid handling rather than enhanced physiologic secretory activity.

Functionally, previous studies have shown that TCs regulate epithelial secretion in other organs. For instance, in mouse intestine and trachea, TCs regulate fluid secretion by releasing acetylcholine onto neighboring epithelial cells, which activates Cl^−^ channels to drive chloride and water secretion into the lumen [90,91]. Moreover, TCs reprogram other epithelial cells to modify antimicrobial protein secretion in the small intestine [92]. These observations across mucosal organs support a broader role for TCs as key modulators of epithelial secretory programs. In SGs, our findings demonstrate that TC loss is associated with saliva quantity and quality dysregulation. Specifically, saliva flow rates from female and male C57BL/6J-*Pou2f3^-/-^* mice were altered when compared with wild-type controls and saliva proteomics revealed sex-divergent changes in protein composition. Together, TC loss is associated with alterations in saliva quantity and quality in a sex-specific manner and further studies are warranted to determine how these changes impact oral cavity integrity.

Prior work has shown that TCs are rare epithelial cells in human SGs [37,39]. In SG disease, previous studies showed that TCs are enriched in benign neoplasms such as Warthin tumors and pleomorphic adenomas, whereas malignant tumors contain only scattered TCs [39]. Moreover, TCs in Warthin tumors show higher levels of hematopoietic prostaglandin D synthase and produce more prostaglandin D_2_ (PGD_2)_ when compared with TCs in healthy striated ducts, suggesting that the PGD_2_ released from TCs may be associated with the pathogenesis of this neoplasia [40]. More recently, Zhang et al. reported that DCLK1^+^ cells with TC features are prominent in ducts in minor SGs from SjD patients compared to labial cyst controls, with particularly strong DCLK1 and IL-25 signals in dilated ducts adjacent to lymphoid foci and evidence of DCLK1–IL-25 colocalization [93]. In contrast, TC numbers were heterogeneous in our human minor SG cohort, ranging from biopsies with high counts to samples lacking detectable cells. Moreover, our data indicate that TC numbers in human minor SGs shift with the presence and severity of glandular injury and autoimmunity, although it remains unclear whether these changes contribute to or simply reflect salivary gland pathology. Importantly, differences across our findings and Zhang’s may reflect: (a) non-equivalent comparators (nSjD vs. labial gland cyst as controls), (b) marker of choice (POU2F3, a TC-lineage regulator, versus DCLK1, which is considered to be a reliable TC marker in rodents, but not in humans [93,94,95], and (c) measurement approaches (we used area-normalized TC counts, whereas Zhang et al. report DCLK1^+^ staining patterns without clearly stating an area-normalized density metric). These discrepancies highlight the need for larger, well-stratified cohorts with standardized markers and quantification. Finally, TC numbers are also altered in other inflammatory conditions. For instance, in the intestine from human patients with Crohn’s disease and from a Crohn’s-like disease mouse model, TCs are significantly reduced when compared with non-disease controls [56]. In contrast, in human chronic rhinosinusitis, TC numbers are increased in nasal polyp tissue compared with adjacent non-polypoid sinonasal mucosa [57]. Together, these findings indicate that TC numbers are altered in human SG inflammation, a pattern we also observed in minor SGs from our SjD patients. Therefore, mechanistic studies are warranted to determine whether TCs are the cause or consequence of the autoimmune environment in SG inflammation.

This study has limitations that should be acknowledged. First, we used a global *Pou2f3* knockout, which eliminates TCs in multiple organs and may introduce systemic effects. Thus, salivary-specific conditional deletion will be essential to dissect local versus systemic contributions. Second, our transcriptomic and proteomic analyses are descriptive and targeted functional experiments will be required to pinpoint the critical TC-dependent pathways. Third, our minor SG samples were drawn from SjD patients with focus scores ranging from 1.06 to 7.06; however, the heterogeneity of disease severity in this limited cohort restricts our ability to relate our data to disease activity and progression. Therefore, a systematic evaluation of TCs in healthy minor SGs and in larger SjD cohorts, with samples stratified by focus score, is warranted to determine how TC numbers track with disease activity and progression.

## 5. Conclusions

In summary, our data suggest that TCs are conserved epithelial sentinels for SG integrity in mice and humans. In mouse SMGs, TC loss is associated with sex-specific transcriptomic, architectural and functional remodeling, whereas in human SGs TC numbers correlate with key clinical, serologic and histopathologic features of SjD. Together, these findings bring three central questions into focus: How do TCs modulate saliva secretion? Are changes in TC numbers a driver or a consequence of SG autoimmune injury? Can TC signaling pathways be targeted to restore secretory function and reduce inflammation? Therefore, addressing these questions in future studies will be essential to translate TC biology into next-generation diagnostic and therapeutic strategies for SG disorders.

## Figures and Tables

**Table 1:** Quantitative analysis of TC density in minor salivary gland (MSG) biopsies. The table reports group size (n), and POU2F3^+^ TC density (mean ± SD and range; cells/mm^2^ of salivary gland area). TC-absent samples were observed in 3/20 nSjD cases and 8/40 SjD cases, indicating inter-individual heterogeneity. Cohort sizes: nSjD, n = 20; SjD, n = 40.

Group	n	Mean ± SD (POU2F3^+^ TCs/mm^2^)	Range of POU2F3^+^ TCs/mm^2^
nSjD	20	2.76 ± 3.01	0–9.24
SjD	40	3.27 ± 4.73	0–23.94

## Data Availability

The bulk RNA-seq data generated in this study have been deposited in the NCBI Gene Expression Omnibus (GEO) under accession number GSE320144. Saliva proteomics raw and processed data are available via ProteomeXchange with identifier PXD075581. Other data supporting the findings of this study are available within the article/Supplementary Materials and its Appendix A. Finally, additional data will be made available upon request.

## Supplementary Materials

The following supporting information can be downloaded at: https://www.mdpi.com/article/10.3390/cells15070583/s1, Table S1. Significantly enriched Ingenuity Pathway Analysis pathways and contributing DEGs in female *Pou2f3^-/-^* vs. wild-type submandibular gland. Ingenuity Pathway Analysis results showing significantly enriched canonical pathways and the contributing DEGs identified in female *Pou2f3^-/-^* vs. wild-type submandibular salivary glands. Table S2. Significantly enriched Ingenuity Pathway Analysis pathways and contributing DEGs in male *Pou2f3^-/-^* vs. wild-type submandibular gland. Ingenuity Pathway Analysis results showing significantly enriched canonical pathways and the contributing DEGs identified in male *Pou2f3^-/-^* vs. wild-type submandibular salivary glands. Table S3. Demographic, clinical, and histopathological features of individuals with Sjögren’s disease and non-Sjögren sicca. Data are presented for the study cohorts and include the variables used for clinical and histopathological characterization. Table S4. DEGs in female and male *Pou2f3^-/-^* mice. Lists of DEGs identified in female and male *Pou2f3^-/-^* mice compared to wild-type controls, including fold change and statistical significance metrics. Figure S1. Distribution of focus scores in minor salivary gland samples from the Sjögren’s disease and non-Sjögren sicca groups. The black horizontal line indicates the median focus score for each group.

## Abbreviations

The following abbreviations are used in this manuscript:

ACR/EULAR	American College of Rheumatology/European League Against Rheumatism
ANA	Antinuclear antibody
CI	Confidence interval
DEG/DEGs	Differentially expressed gene(s)
FDR	False discovery rate
IL-25	Interleukin-25
KRT8	Keratin 8
PGD2	Prostaglandin D2
POU2F3	POU class 2 homeobox 3
RNA-seq	RNA-sequencing
RR	Rate ratio
SG/SGs	Salivary gland(s)
SjD	Sjögren’s disease
SMG/SMGs	Submandibular gland(s)
STRING	Search Tool for the Retrieval of Interacting Genes/Proteins
TC/TCs	Tuft cell(s)
TEM	Transmission electron microscopy

## Appendix A

**Table A1:** Poisson regression model for counting of TCs considering gland area (mm^2^) as a covariate.

Group	RR [95% CI]	*p*-Value	Adjusted R^2^
SjD	1	<0.001	0.376
NSjD	1.12 [1.02–1.23]		
Clinical aspects			
UWS (mL/min)			
>0.1	1	0.005	0.379
≤0.1	0.82 [0.75–0.89]		
Laboratory exams			
Anti-SSA/Ro			
Positive	1	<0.001	0.447
Negative	2.07 [1.85–2.32]		
Anti-SSB/La			
Negative	1	<0.001	0.457
Positive	0.38 [0.33–0.44]		
Antinuclear antibody			
Positive	1	<0.001	0.466
Negative	3.27 [2.86–3.73]		
Rheumatoid factor			
Negative	1	0.079	0.391
Positive	1.10 [0.99–1.21]		
Histopathological aspects			
Focus Score			
≥1	1	0.018	0.376
<1	1.12 [1.02–1.23]		
Germinal centers			
No	1	<0.001	0.450
Yes	0.19 [0.15–0.24]		
Lymphoepithelial lesion			
Yes	1	<0.001	0.394
No	0.60 [0.54–0.67]		
Inflammation *			
Present	1	>0.05	0.377
Absent	1.07 [0.97–1.18]		
Acinar atrophy			
Present	1	<0.001	0.389
Absent	0.69 [0.62–0.76]		
Acinar dilatation			
Absent	1	0.001	0.379
Present	1.35 [1.12–1.63]		
Ductal dilatation			
Present	1		0.383
Absent	0.76 [0.69–0.83]		
Fibrosis			
Absent	1	<0.001	0.395
Present	1.66 [1.48–1.85]		
Adipose replacement			
Absent	1	<0.001	0.405
Present	1.73 [1.57–1.91]		

RR, rate ratios; CI, confidence interval, SjD, Sjögren’s Disease; UWS, Unstimulated Whole Saliva. * Inflammation represents the infiltration of inflammatory cells dispersed and in clusters in preserved and non-preserved glandular tissue. Inflammation was scored as present and absent.
