# Supplementary material for: Highlights of Tuft Cells in Mouse and Human Salivary Glands
Source: Cells. 2026 Mar 25;15(7):583. doi: 10.3390/cells15070583 (PMC13072126; doi:10.3390/cells15070583)
Supplement: Supplementary file 1 [file cells-15-00583-s001.zip › Table S3.pdf]

Supplementary Table S3. Demographic, clinical, and histopathological features of individuals with Sjögren Disease and Non-Sjögren Sicca.

	SjD	nSjD	p-value
**Sex**			
Female	40 (69.0)	18 (31.0)	**p<0.05***
Male	0 (0.0)	2 (100.0)	
**Age – Median (range)**	60 (29 – 76)	59 (29 – 79)	p>0.05**
**Alcohol**			
Yes	5 (83.3)	1 (16.7)	p>0.05*
Never	27 (61.4)	17 (38.6)	
Interrupted	3 (100.0)	0 (0.0)	
NR	5 (8.3)	2 (3.3)	
**Tabacco**			
Yes	2 (66.7)	1 (33.3)	p>0.05*
Never	25 (67.6)	12 (32.4)	
Interrupted	5 (62.5)	3 (37.5)	
NR	8 (13.3)	4 (6.6)	
**Disease duration (y) – Median (range)**	1.5 (0 - 18)		
**Rheumatoid arthritis**			
Yes	3 (30.0)	7 (70.0)	**p<0.01***
No	36 (78.3)	10 (21.7)	
NR	1 (1.66)	3 (5.0)	
**Systemic lupus erythematosus**			
Yes	2 (66.7)	1 (33.3)	p>0.05*
No	37 (69.8)	16 (30.2)	
NR	1 (1.66)	3 (5.0)	
**Medication**			
Yes	37 (67.3)	18 (32.7)	p>0.05*
No	3 (75.0)	1 (25.0)	
**Antidepressant**			
Yes	19 (65.5)	10 (34.4)	p>0.05*
No	20 (69.0)	9 (31.0)	
**Antihypertensive**			
Yes	23 (59.0)	13 (65.0)	p>0.05*
No	16 (41.0)	7 (35.0)	
Clinical aspects			
**Xerostomia**			
Yes	36 (67.9)	17 (32.1)	**p<0.05***
No	2 (40.0)	3 (60.0)	
NR	2 (3.33)	0 (0.0)	

**Xeropthalmia**			
Yes	25 (69.4)	11 (30.6)	**p<0.05***
No	4 (36.4)	7 (63.9)	
NR	11 (18.3)	2 (3.3)	
**Parotitis**			
Yes	13 (81.3)	3 (18.8)	p>0.05*
No	22 (61.1)	14 (38.9)	
NR	5 (8.3)	3 (5.0)	
**UWS - Median (range)**	0.06 (0.0 – 0.6)	0.15 (0.04 – 0.8)	**p<0.01****
**UWS**			
≤0.1 ml/min	28 (84.8)	5 (15.2)	**p<0.01***
>0.1 ml/min	12 (44.4)	15 (55.6)	
Laboratory exams			
**Anti - SSA**			
Positive	17 (89.5)	2 (10.5)	**p<0.01***
Negative	21 (53.8)	18 (46.2)	
NR	2 (3.33)	0 (0.0)	
**Anti-SSB**			
Positive	10 (90.9)	1 (9.1)	p>0.05*
Negative	27 (60.0)	18 (40.0)	
NR	3 (5.0)	1 (1.66)	
**Rheumatoid Factor**			
Positive	16 (88.9)	2 (11.1)	**p<0.05***
Negative	17 (58.6)	12 (41.4)	
NR	7 (11.6)	6 (10.0)	
**Antinuclear antibody**			
Positive	22 (81.5)	5 (18.5)	**p<0.05***
Negative	12 (50.0)	12 (50.0)	
NR	6 (10.0)	3 (5.0)	
Histopathological aspects			
**Focus score – Median (range)**	2.9 (1.0 – 7.0)	0.6 (0.0 – 0.9)	**p<0.01****
**Focus score**			
≥1	40 (100.0)	0 (0.0)	**p<0.01***
<1	0 (0.0)	20 (100.0)	
**Number of histological focus – Median (range)**	8.5 (3.0 – 29.0)	2.0 (0.0 – 6.0)	**p<0.01****
**Germinal centers**			
Present	4 (100.0)	0 (0.0)	p>0.05*
Absent	36 (64.3)	20 (35.7)	
**Lymphoepithelial lesion**			
Present	8 (100.0)	0 (0.0)	**p<0.05***
Absent	32 (61.5)	20 (38.5)	

**Inflammation**			
Present	35 (94.6)	2 (5.4)	**p<0.01***
Absent or very low	5 (22.7)	17 (77.3)	
**Acinar atrophy**			
Present	19 (70.4)	8 (29.6)	p>0.05*
Absent or very low	21 (63.6)	12 (36.4)	
**Acinar dilatation**			
Present	2 (66.7)	1 (33.3)	p>0.05*
Discreet or very low	38 (67.9)	18 (32.1)	
**Ductal dilatation**			
Present	12 (70.6)	5 (29.4)	p>0.05*
Absent or very low	28 (65.1)	15 (34.9)	
**Fibrosis**			
Present	9 (60.0)	6 (40.0)	p>0.05*
Absent or very low	31 (68.9)	14 (31.1)	
**Adipose replacement**			
Present	10 (58.8)	7 (41.2)	p>0.05*
Absent or very low	30 (69.8)	13 (30.2)	

*Chi-square test

**Mann-Whitney U

SjD, Sjogren Disease; nSjD, Non-Sjögren Sicca; SD, standard deviation; y, years; Bolds denotes statistical significance; UWS, Unstimulated Whole Saliva
