# Supplementary figures and images for: Highlights of Tuft Cells in Mouse and Human Salivary Glands
Source: Cells. 2026 Mar 25;15(7):583. doi: 10.3390/cells15070583 (PMC13072126; doi:10.3390/cells15070583)

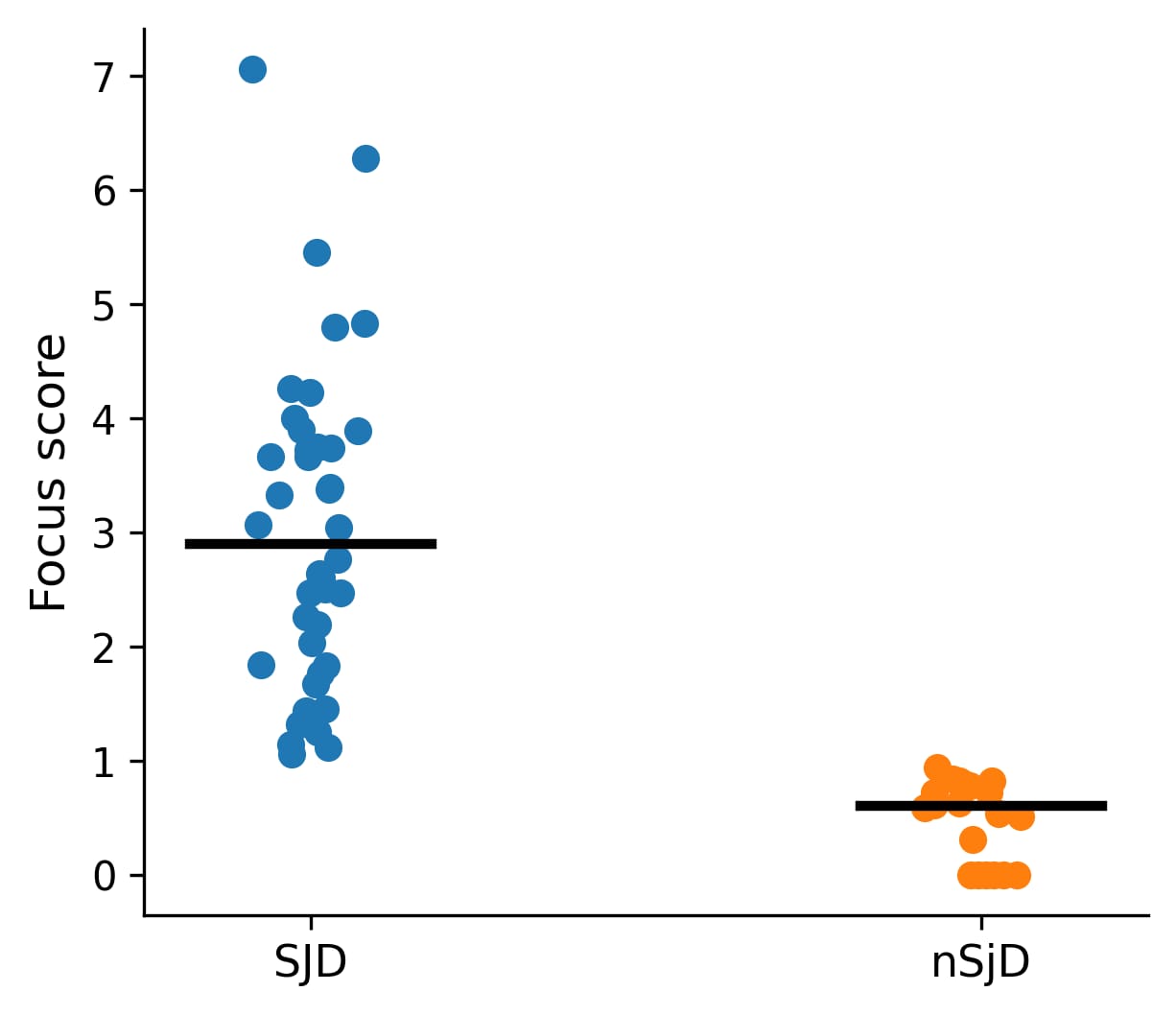

Supplement: Supplementary file 1 [file cells-15-00583-s001.zip › Figure S1.jpeg]
